# In-Hospital Levels of Circulating MicroRNAs as Potential Predictors of Left Ventricular Remodeling Post-Myocardial Infarction

**DOI:** 10.3390/medicina60010149

**Published:** 2024-01-13

**Authors:** Michał Węgiel, Marcin Surmiak, Krzysztof Piotr Malinowski, Artur Dziewierz, Andrzej Surdacki, Stanisław Bartuś, Tomasz Rakowski

**Affiliations:** 1 Clinical Department of Cardiology and Cardiovascular Interventions, University Hospital in Krakow, 30-688 Krakow, Poland; michal.jan.wegiel@gmail.com (M.W.); adziewierz@gmail.com (A.D.); stanislaw.bartus@uj.edu.pl (S.B.); 2 Department of Internal Medicine, Jagiellonian University Medical College, 30-688 Krakow, Poland; 3 Department of Bioinformatics and Telemedicine, Jagiellonian University Medical College, 30-688 Krakow, Poland; 4 2nd Department of Cardiology, Institute of Cardiology, Jagiellonian University Medical College, 30-688 Krakow, Poland

**Keywords:** microRNA, biomarkers, myocardial infarction, myocardial remodeling

## Abstract

*Background and Objectives*: Biochemical and molecular regulation of both adaptive and pathological responses of heart tissue to ischemic injury is widely investigated. However, it is still not fully understood. Several biomarkers are tested as predictors of left ventricle (LV) remodeling after myocardial infarction (MI). The aim of this study was to assess the relationship between selected microRNAs (miRNAs) and LV function and morphology in patients after MI. *Materials and Methods*: Selected miRNAs related to heart failure were assessed in the acute phase of MI: miR-150-3p, miR-21-5p, miR-19b-3p, miR-155-5p, miR-22-5p. Echocardiography with 3D imaging was performed at baseline and after 6 months. Remodeling was defined as >20% increase in LV end-diastolic volume, whereas reverse remodeling was defined as >10% reduction in LV end-systolic volume. *Results*: Eighty patients entered the registry. Remodeling occurred in 26% and reverse remodeling was reported in 51% of patients. In the presented study, none of the analyzed miRNAs were found to be a significant LV remodeling predictor. The observed correlations between miRNAs and other circulating biomarkers of myocardial remodeling were relatively weak. *Conclusions*: Our analysis does not demonstrate an association between the analyzed miRNAs and LV remodeling in patients with MI.

## 1. Introduction

Myocardial infarction (MI) is a complex clinical syndrome which could be considered in aspects of health, economical, and sociological burden [1]. Cardiovascular diseases continue to be the leading cause of death worldwide. In particular, ischemic heart disease is considered as a main cause of mortality globally. Primary percutaneous coronary intervention (PCI), intensive cardiac care and potent antiplatelet agents constitute a decreasing acute mortality due to MI [1]. Nowadays, more patients survive MI. However, the impact of myocardial ischemia translates far beyond the acute phase. Neurohormonal activation and inflammatory reactions after ischemia could lead to myocardial remodeling, which is gradual changes in left ventricle (LV) morphology, geometry, and function. This could result in, at first, asymptomatic, but later clinically apparent, myocardial dysfunction and heart failure [2,3,4]. The latter is associated with high mortality as well as frequent re-hospitalizations, prolonged hospital stay and disability [2,3,4]. For this reason, early identification of high-risk individuals is crucial. On the other hand, due to early revascularization therapy and targeted pharmacotherapy, in some patients, improvement of an initially impaired heart muscle is observed during follow up. This phenomenon is described as reverse remodeling [5]. The biochemical and molecular regulation of both adaptive and pathological responses of heart tissue to ischemic injury is widely investigated, though it remains incompletely understood. Several biomarkers are under investigation as potential predictors of LV remodeling post-MI, including those linked to inflammatory responses, fibrosis, cardiac stretch, and cell proliferation [5,6]. Despite extensive research, selecting the optimal biomarker remains challenging [5,6,7,8,9].

MicroRNAs (miRNAs) are small, single-stranded, non-coding molecules that regulate gene expression and physiological as well as pathological processes [10]. Circulating miRNAs are released into the blood and thus are potentially available as markers of several diseases. MicroRNAs are implicated in various cardiovascular disorders including atherosclerosis, atrial fibrillation, MI, and heart failure [10,11,12]. Recently, emerging data have revealed their role in post-MI cardiac remodeling [13]. In the literature, specifically that of Polish origin, several previous studies focused on the association between different miRNAs and MI and myocardial remodeling, and yet there is still no consensus as to which molecules could act as clinically useful biomarkers [13,14,15]. Thus, we sought to assess the relationship between circulating microRNAs and changes in LV function and morphology in patients who have experienced MI. Due to previous reports about their association with cardiovascular system, the following miRNAs were selected: miR-150-3p, miR-21-5p, miR-19b-3p, miR-155-5p, miR-22-5p.

## 2. Materials and Methods

### 2.1. Study Design

The present study was performed on a previously analyzed group of 80 patients who had suffered acute MI and were treated with primary PCI. The study’s population and design have been elaborately described in our previous report [5]. To summarize, it was a prospective analysis aimed at identifying predictors of post-MI LV remodeling. ST-elevation and non-ST-elevation MI patients were included; however, only patients with an immediate invasive strategy with primary PCI performed in the first 2 h of admission and with culprit lesion located in a major coronary artery were analyzed. The main exclusion criteria were a history of previous MI, baseline LV ejection fraction < 25%, and cardiogenic shock at admission. A summary of the baseline patient characteristics can be found in Supplementary Material Tables S1 and S2.

The analysis aimed to assess selected miRNAs in the acute MI setting and evaluate associations between the levels of these circulating molecules and the morphology and function of LV during the acute phase of MI and after 6 months of follow-up. The following miRNAs were selected: miR-150-3p, miR-21-5p, miR-19b-3p, miR-155-5p, miR-22-5p. The role of these molecules in the regulation of the cardiovascular system and especially in MI, heart failure, and myocardial remodeling is briefly summarized in Table 1.

### 2.2. Imaging Assessment

Imaging assessment was reported in detail in our previous work [5]. Patients underwent transthoracic echocardiographic examination with 3D imaging on the 2nd–3rd day of hospitalization and again after a 6-month follow-up. These examinations were conducted to assess the occurrence of adverse remodeling (defined as an increase in LV end-diastolic volume by more than 20%) and reverse remodeling (defined as a reduction in LV end-systolic volume of more than 10%). Echocardiography images were recorded using the Vivid E9 ultrasound machine with 3D imaging (General Electric, Boston, MA, USA). Remodeling analysis was performed offline and conducted by an independent investigator with usage of EchoPac software (Version: 113,General Electric, USA). Volumes of LV were calculated using the Simpson triplane method from 3D scans [16].

### 2.3. Blood Sampling and Molecular Analysis

The procedures for blood sampling, preparation, and biochemical analyses have been detailed in our previous publication [5]. Blood samples were obtained via single collection from the peripheral vein during the acute phase of MI (2nd–3rd day after hospital admission) using Sarstedt S-Monovette tubes. Samples were centrifuged at 3000 rpm for 15 min, and then plasma was aliquoted and frozen at −80 °C.

In the current analysis, total RNA (including miRNA) was isolated from serum and cell culture medium, which was collected post-monocyte stimulation. This isolation was carried out using a commercial kit (mirVana™ PARIS™ RNA and Native Protein Purification Kit, Invitrogen, Carlsbad, CA, USA) and the RNA was reverse transcribed according to the manufacturer’s instructions (TaqMan™ Advanced miRNA cDNA Synthesis Kit, Thermo Fisher Scientific, Waltham, MA, USA). The quantification of selected miRNAs was accomplished in all study participants using qRT-PCR and TaqMan chemistry-specific assays (QuantStudio 12K Flex Real-Time PCR System; Life Technologies, Carlsbad, CA, USA). The results were calculated using the 2^−ΔCt^ (relative expression, RE) formula based on the exogenous internal control (cel-39 miRNA spike-in).

Previously analyzed biomarkers which we correlated with the present miRNA were assessed using the MAGPIX fluorescent-based detection system and Luminex assays (galectin-3, MR-proADM, Notch-1, myeloperoxidase, sST2 and syndecan-4). The electrochemiluminescence immunoassay method was used to measure levels of IL-6 and S-100 protein. The spectrophotometric method was used to measure levels of LDH. The latex particle-enhanced immunoturbidimetric method was used to measure CRP levels. Details of the laboratory analysis of these biomarkers were presented in our previous work [5].

### 2.4. Statistical Analysis

Quantitative variables were summarized using either means and standard deviations (for normally distributed data) or medians, along with the first and third quartiles (for non-normally distributed data). Categorical variables were presented as percentages. Due to the low detection rates of miR-150-3p, miR-155-5p, and miR-22-5p, we categorized the results of these molecules as categorical variables. The normality of data was evaluated using the Shapiro–Wilk test. Comparisons of continuous variables were conducted using either the paired t-test (for normally distributed data) or the Mann–Whitney U test (for non-normally distributed data). The chi-square test was used to compare categorical data. A logistic regression analysis was constructed to identify adverse and reverse remodeling predictors. All analyzed miRNAs were considered, and forward selection with a probability value for covariates to enter the model was set at the 0.05 level. The results are presented as odds ratios (OR) with an associated 95% confidence interval (CI). Correlations between microRNAs and other circulating biomarkers were calculated using the Spearman test. A statistical significance threshold was set at an alpha value of <0.05. All statistical analyses were performed using SPSS Statistics 28 (IBM, Inc., Armonk, NY, USA).

## 3. Results

As reported in our previous analysis, most patients after MI experienced improvement in LV function during follow up [5]. Adverse remodeling was observed in 26% of patients, while reverse remodeling was noted in 52% of patients [5]. As previously described, there were no significant differences in demography and distribution of ST-elevation and non-ST-elevation MI between analyzed groups of patients regarding the occurrence of adverse or reverse remodeling (Tables S1 and S2). However, patients with adverse remodeling more commonly had diabetes mellitus and more frequently had distal embolization in angiography and persistent ST elevations in ECG, without resolution during hospital stay (Table S1). On the contrary, patients who developed reverse remodeling less frequently had completely occluded infarct-related arteries in angiography (Table S2). Pharmacological treatment at discharge was also analyzed including antiplatelet- and heart failure-focused therapy (beta-blockers, angiotensin-converting enzyme inhibitors/angiotensin receptor blockers, mineralocorticoid receptor antagonists, diuretics). No statistically significant associations were found regarding the occurrence of adverse and reverse remodeling (Tables S1 and S2).

In relation to the current analysis, Table 2 and Table 3 present the levels of selected miRNAs during the acute phase of MI in relation to the occurrence of adverse and reverse remodeling, respectively. We found no significant differences in the levels of miR-21-5p and miR-19b-3p or the percentage detection of miR-150-3p, miR-155-5p, and miR-22-5p between patients with and without adverse remodeling (Table 2). Similar results were obtained for the occurrence of reverse remodeling (Table 3). In Table 4, we present the logistic regression analysis aimed at predicting adverse and reverse remodeling. In our study, none of the analyzed miRNAs reached statistical significance as a predictor of both types of remodeling. Table 5 shows correlations between miRNAs and previously assessed biomarkers measured during the acute phase of MI. Single correlations between analyzed miRNAs and inflammatory, cardiac stretch, and cell proliferation biomarkers were found, including mid-regional pro-adrenomedullin, NOTCH-1, S-100 protein, and ST2.

## 4. Discussion

Acute mortality after MI is constantly decreasing due to cath lab networks, intensive cardiac care, and targeted pharmacotherapy [1]. Nowadays, more patients survive MI. However, long-term follow-up of these patients is extensively evaluated as the prevalence of ischemia-induced heart failure is increasing. Heart failure remains one of the main complications after MI. The main cause of heart failure is adverse remodeling after MI [2,3,4]. The obstruction of epicardial coronary arteries and ischemia forces cardiomyocytes to develop an anaerobic metabolism with destabilization of the cell membrane, leading to cell death. Massive destruction of cardiac cells leads to an excessive immune response. Influx of neutrophils and macrophages into the infarct zone causes the destruction of the extracellular collagen matrix [17]. This could lead to thinning, dilatation, and the expansion of the infarcted area. Later, fibroblasts are directed into the infarcted zone, where they create scar tissue. The inflammation may persist after MI due to the continuous exposure to parietal stress. The activation of two major neurohormonal systems, the renin–angiotensin–aldosterone system and the sympathetic nervous system, intensifies the apoptotic changes. In the late phase of post-MI remodeling, which takes place approximately one month after the index event, a remote area to the initial infarction becomes affected. The diminished amount of properly functioning myocardial segments in conditions of increased workload causes wall stress. This affects myocytes that are viable yet become adaptively hypertrophied and dilated. Overstretched cardiomyocytes finally lose their compensatory mechanism and eventually lead to LV dilation [17].

The inability of adult cardiomyocytes to proliferate in response to stress and injury after MI is a major trigger of adverse myocardial fibrosis. The loss of viable myocardium due to ischemia initiates fibrotic processes and scar formation, which, on the other hand, in the early phase after MI, provides stabilization of the heart. The production of extracellular matrix components is essential to provide mechanical support to the regenerating heart after cardiac injury. However, the dynamic change in extracellular matrix production forces the heart tissue into maladaptive repair, prolonged inflammatory response, and loss of cardiomyocytes, which ultimately leads to cardiac remodeling. In acute MI, the loss of sustaining tissue makes this area more susceptible to deformation, thinning, and dilation of the cavity. This increases the likelihood of myocardial rupture and is an anatomical substrate for aneurysms. Activated immune cells and fibroblasts protect against myocardial stress by patching the area of damage with a fibrotic scar. Adverse left ventricular remodeling is observed in roughly a quarter of patients post-MI [17]. A consensus about cardiac remodeling was set which defines this phenomenon as a group of molecular, cellular, and interstitial changes that clinically manifest as changes in the size, shape, and function of the heart resulting from cardiac injury [17]. The clinical diagnosis of remodeling is established based on the results of imaging examinations, mainly echocardiography and cardiac magnetic resonance. In imaging modalities, changes in the cavity volumes, mass, geometry, areas of scar after MI, and fibrosis are assessed. The gold standard for heart chamber quantification is cardiac magnetic resonance. However, in every day clinical practice, echocardiography is the most common imaging modality due to its wide availability and ease of use as a bedside examination in intensive cardiac care or even cath lab [16]. Pathological changes in the heart tissue triggered by ischemia can lead to myocardial dysfunction, generally associated with enlargement of the LV cavity and reduced contractility resulting in at first asymptomatic and then later clinically evident myocardial pathology and chronic heart failure. Ischemia is the main cause of heart failure. This condition is a global health concern, associated with high mortality, and is the most common reason for hospital admissions among patients over 65 years of age [18]. Previous studies reported that approximately 50% of patients with heart failure die within 5 years [18]. Cardiac fibrosis results not only in reduced contractility and heart failure but also impairs electrical conduction and reentry and may trigger ventricular arrhythmias.

Extensive research was performed aimed at methods of prognosis and diagnosis of early stages of myocardial dysfunction, as well as efficient therapeutics for cardiac repair. Despite the availability of heart failure drugs that interact with general pathways involved in myocardial remodeling, targeted drugs remain absent. In experimental studies, various agents and molecules have been tested to extrinsically induce regeneration of heart tissue after MI. However, the results of these studies are still in the field of experimental/low-volume research and lack adequate success to be used in real-life clinical situations [13]. On the contrary, some individuals recover from initial myocardial dysfunction post-MI [19]. Such a phenomenon is often described as reverse remodeling. This term previously referred to patients with chronic heart failure responding to resynchronization therapy. However, as we report in this study and in our previous analysis, some patients after MI with initial LV dysfunction experience improvements in LV systolic function and geometry [5]. Consequently, early identification of patients in relation to the likelihood of LV adverse and reverse remodeling is crucial. Biomarker analysis presents a promising strategy due to its non-invasive nature, wide availability, and operator independence. The idea of biomarker testing and early identification of high-risk individuals in order to apply tailored management is very attractive. Tailored, specific interventions might include targeted therapy, as well as more frequent screening for detecting early stages of various diseases [6]. Numerous studies have attempted to identify circulating biomarkers as predictors of LV function post-MI [5,6,7,8,9]. Nevertheless, due to the complex character of post-MI remodeling, which involves mechanical, neuroendocrine, and molecular pathways, selecting an optimal biomarker that reflects multiple pathological mechanisms remains demanding. Despite numerous studies, in real-life scenarios we still do not have clinically useful biomarkers of prognosis after acute MI apart from cardiac troponin and natriuretic peptides [6,7].

MicroRNAs have recently emerged as promising cardiovascular biomarkers [10]. These small, non-coding molecules regulate post-transcriptional gene expression and thus affect a broad range of biological processes, including cell proliferation, metabolism, apoptosis, and immune responses [11]. They consist of 21–23 nucleotides. They are abundant in many cell types and as extracellular circulating miRNAs. MicroRNAs appear to target about 60% of the genes of humans and other mammals, and many miRNAs are evolutionarily conserved, which confirms that they are associated with important biological functions [10]. The cellular role of miRNA is connected with gene regulation. Just as miRNA is involved in the normal functioning of cells, dysregulation of miRNA been associated with various diseases. Several miRNAs are released into circulation and are expressed in heart tissue during MI [10]. In the context of post-MI LV remodeling and subsequent heart failure, the role of miRNAs is linked with the regulation of fibrotic processes, cardiac cell apoptosis, and inflammation [11]. Thus, miRNAs are considered to be a central part of the development of various cardiac disorders [11,12]. However, the effect of miRNA on myocardial remodeling is still not fully understood. Analyzing the impact of individual miRNAs is a challenge. The effect of these molecules is often multi-directional and ambiguous, including modulating both pro- and anti-fibrotic processes through transforming growth factor beta signaling pathways. Time-dependent balanced activation of miRNA after MI is responsible for a favorable outcome after MI. On the other hand, dysregulation of various miRNAs promotes development of cardiac fibrosis [13]. The latter is characterized by excessive extracellular matrix deposition and impairs both systolic and diastolic function of the heart. In numerous previous reports, several miRNAs have been examined. In Table 1, we show a brief summary of selected miRNAs and their role in myocardial remodeling.

MicroRNA-21is a multifunctional molecule with various roles in the cardiovascular system. Although miR-21 is one of the most assessed microRNAs, and its impact on the cardiovascular system is well established, its role as a biomarker of myocardial remodeling remains unclear. Several studies presented inconclusive reports about the role of miR-21 in post-MI remodeling [20,21,22,23]. MiR-21 is expressed in fibroblasts and promotes fibrotic processes by affecting the transforming growth factor beta pathway. It is associated with pro-fibrotic activity and hypertrophy [20]. Previous studies have demonstrated elevated miR-21 expression during MI and heart failure [21]. A higher concentration of circulating miR-21 was reported to be a predictor of post-MI remodeling [22]. Experimental studies showed that inhibiting miR-21 promotes beneficial effects in heart failure. On the other hand, other studies showed the protective effect of miR-21 against inflammation, apoptosis, and cardiac dysfunction after MI, as well as its protective role against hypoxia. In animal models, transgenic mice with miR-21 over-expression demonstrated a reduction in the infarct area and fibrosis [23].

MicroRNA-22 is highly expressed in the heart. In fact, it is the most abundant microRNA in heart tissue. It is up-regulated during the acute phase of MI and correlates with levels of cardiac troponin I. It is an essential component of cardiac reactions to stress, involving hypertrophy, fibrosis, sarcoma reorganization, and apoptosis [24]. MicroRNA-22 has been demonstrated to regulate cardiac hypertrophy. A previous report has shown the prognostic value of miR-22 levels on the occurrence of post-MI remodeling. Experimental studies showed that overexpression of miR-22 leads to cardiac hypertrophy. On the other hand, in miR-22 knockout models exposed to stress, cardiac dilation and fibrosis were observed [24]. In animal models, those with miR-22 deficiency had pathological reactions to cardiac stress and, instead of adaptive hypertrophy, presented adverse myocardial remodeling [24].

Several studies reported that miR-155 is closely associated with the cardiovascular system. MicroRNA-155 was indicated as a mediator of cardiac injury and inflammation and as a pro-hypertrophic factor. MicroRNA-155 induces a myofibroblast phenotype in cardiac isolated fibroblasts. Myofibroblasts are the main source of the extracellular matrix and have a fundamental role in cardiac fibrosis [25]. Previous research has shown that miR-155 is up-regulated in patients with LV remodeling post-MI. Other studies showed that inhibition of miR-155 attenuates myocardial remodeling and that failed down-regulation of this molecule promotes remodeling after MI [26]. In other reports, miR-155 has been studied in coronary artery disease [25]. The results of miRNA-155 were contradictory with either the promotion or prevention of the pathophysiological process of atherosclerosis. This confirms the complexity of this molecule [26].

Several miRNAs have been reported to exhibit cardio-protective effects. Animal models have shown that the injection of miR-19 stimulates cardiac regeneration post-MI [27]. Experimental studies showed that miR-19 promotes cardiac cell survival and proliferation in repose to cardiac injury, as well as repressed immune response in the heart. MicroRNA-19 was reported to inhibit fibrosis, as well as regulate hypoxia. Early cardiac protection brings therapeutic promise to treat MI and heart failure. On the other hand, decreased miR-19 was associated with greater risk of heart failure [28].

The up-regulation of miR-150 may inhibit cardiac fibrosis and hypertrophy by regulating fibroblasts [29]. In experimental studies, miR-150 attenuated myocardial remodeling by directly interacting with a myocardial infarction-associated transcript, which, on the other hand, is over-expressed in failing hearts and worsens cardiac remodeling [29]. Low miR-150 levels were associated with LV remodeling after MI. Down-regulation of miR-150 was also reported in other cardiovascular diseases including atrial fibrillation or dilated cardiomyopathy. Other research has linked the cardio-protective effect of miR-150, with its inhibitory effects on C-reactive protein and adrenergic receptor beta 1, as well as in the suppression of pro-apoptotic genes [30,31].

In our study, we did not identify a significant role of the analyzed miRNAs in predicting LV remodeling post-MI. None of the analyzed miRNAs were predictors of LV remodeling. Additionally, the observed correlations between miRNAs and previously analyzed biomarkers were relatively weak.

A previous report has shown that different miRNAs, previously associated with LV dysfunction, fail as biomarkers of LV remodeling after MI [32]. The authors discussed that in this study patients received complete pharmacological treatment for heart failure, as appeased to other reports. This observation applies to our analysis as there was a high rate of administered beta receptor antagonists and angiotensin-converting enzyme inhibitors in the studied population [5]. It is worth mentioning that most studies utilize multiple measurements of miRNA or other biomarkers of myocardial remodeling during the acute phase of MI and further follow-ups. While this strategy provides scientific data, such an approach is far from usual clinical practice, when typically a single time-point measurement is used, like in our study.

Another issue connected with biomarkers of myocardial remodeling is the lack of homogeneity in study design and methodology. First, there is no universal definition of myocardial remodeling or reverse remodeling. Several cut-off values regarding changes in LV end-diastolic and -systolic volume, as well as the LV ejection fraction, are utilized. Second, there is no consensus regarding the time interval from MI to the remodeling assessment. Finally, volumes of LV vary depending on the use of imaging modalities including echocardiography, cardiac magnetic resonance, or computer tomography. Therefore, the results of post-MI studies are, at times, non-overlapping and contradictory [6,7].

### Study Limitations

The presented study has several limitations. The first issue is the relatively small sample size of 80 patients. The second limitation is the single time-point measurement of miRNAs. Unfortunately, no specific data about the pharmacokinetics of the analyzed miRNA are available. Moreover, in previous studies, varying levels of circulating miRNA were reported in different clinical conditions including the revascularization strategy and successful or unsuccessful PCI, which is why it is very challenging to evaluate what is an optimal time-point for microRNA assessment. Possibly, multiple measurements of these molecules are appropriate. Single time-point blood sampling is a limitation of our study. However, our cohort of consecutive MI patients and single time-point measurements reflect a real-world clinical scenario. Usually in typical clinical practice, a single measurement of the biomarker is utilized; yet, as presented in our study, this did not apply to the analyzed miRNAs. Moreover, due to logistic reasons and out of concern for equal scheduling in all patients, the blood samples were collected during the 2nd–3nd day after hospital admission and not during primary PCI, as in some previous reports. However, as previously mentioned, such strategy reflects a more real-life approach as the analyzed molecules do not belong to first-choice assessed parameters during primary PCI in acute MI.

## 5. Conclusions

Our analysis does not demonstrate an association between the analyzed miRNAs and LV remodeling in patients with MI. However, given the relatively small sample size of the current study and the results of previous reports showing the connection of miRNAs with myocardial remodeling, further research involving a larger patient group is necessary.

## Figures and Tables

**Table 1:** Summary of data about role of selected miRNAs in cardiovascular system.

	Role in Cardiovascular System	Acting Mechanism
miR-150-3p	Attenuates myocardial remodeling. Protective role in heart failure. Down-regulated in myocardial remodeling.	Interacting with myocardial infarction-associated transcript. Inhibiting C-reactive protein and adrenergic receptor beta-1. Inhibiting pro-apoptotic genes.
miR-21-5p	Still not clear. Reports about pro-fibrotic activity and also the cardio-protective role against apoptosis.	Expressed in fibroblasts. Increases fibrosis and cardiac hypertrophy.
miR-19b-3p	Still not clear. Reports about pro-fibrotic activity and also the cardio-protective role. Stimulates cardiac regeneration.	Participates in cardiac fibrosis by modulating transforming growth factor beta pathway.
miR-155-5p	Mediator of cardiac injury and inflammation. Up-regulated in myocardial remodeling.	Pro-inflammatory, oncogenic molecule, expressed in activated B, T, and endothelial cells.
miR-22-5p	Regulates cardiac dilation, fibrosis, and hypertrophy.	Over-expression promotes cardiac hypertrophy. On the other hand, miR-22-5p knock-out + cardiac stress leads to fibrosis and dilation.

**Table 2:** MicroRNA levels in patients with and without adverse remodeling.

	Adverse Remodeling (+) N = 21 (26%)	Adverse Remodeling (−) N = 59 (74%)	*p* Value
miR-150-3p, detected	27.23%	28.08%	1.004
miR-21-5p	5.27 (0.74; 512)	7.32 (0.61; 423)	0.937
miR-19b-3p	0.66 (0.13; 2.02)	0.71 (0.09; 0.93)	0.554
miR-155-5p, detected	7.08%	14.33%	0.670
miR-22-5p, detected	47.32%	31.86%	0.361

miR—microRNA.

**Table 3:** MicroRNA levels in patients with and without reverse remodeling.

	Reverse Remodeling (+) N = 42 (52%)	Reverse Remodeling (−) N = 38 (48%)	*p* Value
miR-150-3p, detected	26.23%	28.88%	1.002
miR-21-5p	3.06 (0.46; 462)	12.8 (0.76; 415)	0.469
miR-19b-3p	0.71 (0.08; 0.87)	0.66 (0.11; 1.07)	0.663
miR-155-5p, detected	9.12%	16.03%	0.462
miR-22-5p, detected	26.09%	45.44%	0.127

miR—microRNA.

**Table 4:** Logistic regression analysis aimed at predicting adverse and reverse remodeling.

	OR	95% CI	*p* Value
Adverse remodeling			
miR-150-3p, detected (y/n)	0.935	0.255–3.432	0.921
miR-21-5p	1.000	1.000–1.001	0.214
miR-19b-3p	1.002	0.998–1.006	0.365
miR-155-5p, detected (y/n)	0.439	0.050–3.883	0.463
miR-22-5p, detected (y/n)	1.859	0.574–6.025	0.301
Reverse remodeling			
miR-150-3p, detected (y/n)	0.880	0.297–2.61	0.820
miR-21-5p	1.000	0.999–1.000	0.319
miR-19b-3p	0.996	0.989–1.002	0.192
miR-155-5p, detected (y/n)	0.503	0.110–2.308	0.377
miR-22-5p, detected (y/n)	0.437	0.155–1.236	0.124

miR—microRNA.

**Table 5:** Correlations between miRNAs and selected previously analyzed biomarkers.

	miR-19b-3p	miR-150-3p	miR-22-5p
MR-proADM	0.277 *	0.110	0.253 *
ST2	0.263 *	−0.127	−0.016
S100	0.261 *	0.162	0.101
NOTCH1	0.030	0.224 *	0.110
miR-21-5p	0.729 **	−0.247 *	−0.210
miR-22-5p	−0.009	0.434 **	-
miR-155-5p	−0.035	0.381 **	0.241 *

* p < 0.05; ** *p* < 0.01. MR-proADM—mid-regional pro-adrenomedullin, ST2—suppression of tumorigenicity, miR—microRNA.

## Data Availability

The data presented in this study are available on request from the corresponding author. The data are not publicly available due to privacy reasons.

## Supplementary Materials

The following supporting information can be downloaded at: https://www.mdpi.com/article/10.3390/medicina60010149/s1, Table S1: Baseline characteristics of patients with and without adverse remodeling; Table S2: Baseline characteristics of patients with and without reverse remodeling.
