# Supplementary material for: In-Hospital Levels of Circulating MicroRNAs as Potential Predictors of Left Ventricular Remodeling Post-Myocardial Infarction
Source: Medicina (Kaunas). 2024 Jan 13;60(1):149. doi: 10.3390/medicina60010149 (PMC10819680; doi:10.3390/medicina60010149)
Supplement: Supplementary file 1 [file medicina-60-00149-s001.zip › medicina-2785794-supplementary.pdf]

Article

Circulating microRNAs as potential predictors of left ventricular remodeling post-myocardial infarction.

Michał Węgiel [1], Marcin Surmiak [2], Krzysztof Malinowski [3], Artur Dziewierz [1,4], Andrzej Surdacki [1,4], Stanisław Bartuś [1,4] and Tomasz Rakowski [1,4,*]

Supplementary

	**Adverse remodeling (+) N=21 (26%)**	**Adverse remodeling (-) N=59 (74%)**	***P* value**
Age, years, mean (SD)	61.23 (10)	64.36 (11)	0.403
Male gender, %	65.02	65.27	-
STEMI, %	76.15	71.34	0.650
NSTEMI, %	24.25	29.09	0.653
Arterial hypertension, %	65.45	65.09	-
Diabetes mellitus, %	41.14	15.08	0.021
Smoking, %	53.19	44.34	0.514
Baseline GFR, ml/min/1.73m^2, median (Q1; Q3)	76.02 (65; 90)	90.47 (78; 90)	0.079
**Angiography details and PCI results**			

Left anterior descending culprit, %	64.87	46.27	0.182
Multi vessel disease, %	43.12	33.39	0.534
TIMI flow before PCI,%			0.853
0	59.05	50.17	
1	1: 6.34	8.46	
2	2: 17.52	27.08	
3	3: 17.49	14.87	
TIMI 0 flow before PCI, %	59.34	49.88	0.362
TIMI 3 flow after PCI, %	82.54	92.29	0.290
Slow flow/No reflow, %	18.09	3.99	0.094
Distal embolization, %	18.04	2.07	0.023
Persistent ST elevations at discharge, %	56.29	24.02	0.024
Glycoprotein IIb/IIIa inhibitor, %	52.86	31.06	0.112
Aspiration thrombectomy during PCI, %	52.94	33.47	0.149

Treatment at discharge			
Acetylsalicylic acid, %	100	100	-
Ticagrelor, %	78.96	69.35	0.738
Clopidogrel, %	21.09	31.18	0.743
B-blocker, %	100	100	-
ACEI/ARB, %	92.86	86.43	0.667
MRA, %	29.06	35.09	0.764
Loop diuretic, %	43.21	31.34	0.520
Statin, %	100	100	-

Table S1. Baseline characteristics of patients with and without adverse remodeling.

STEMI-ST elevation myocardial infarction, NSTEMI-nonST elevation myocardial infarction, GFR-glomerular filtration rate, TIMI-thrombolysis in myocardial infaction, ACEI-angiotensin converting enzyme inhibitor, ARB-angiotensin receptor blocker, MRA-mineralocorticoid receptor antagonist

	Reverse remodeling (+) N=42 (52%)	Reverse remodeling (-) N=38 (48%)	*P* value
Age, years, mean (SD)	63.34 (10)	62.90 (11)	0.747
Male gender, %	71.28	58.08	0.292
STEMI, %	71.46	74.04	0.750
NSTEMI, %	28.77	26.08	0.754
Arterial hypertension, %	62.27	68.01	0.612
Diabetes mellitus, %	18.01	26.46	0.420
Smoking, %	46.82	45.08	0.881
Baseline GFR, ml/min/1.73m^2, median (Q1; Q3)	90.21 (75; 90)	89.09 (69; 90)	0.532
**Angiography details and PCI results**			
Left anterior descending culprit, %	47.46	55.06	0.530
Multi vessel disease, %	27.15	43.34	0.204

TIMI flow before PCI,%			0.102
0	38.45	67.79	
1	12.08	3.33	
2	29.02	19.25	
3	20.88	10.02	
TIMI 0 flow before PCI, %	38.46	67.88	0.021
TIMI 3 flow after PCI, %	91.48	87.08	0.623
Slow flow/No reflow, %	3.24	12.89	0.130
Distal embolization, %	2.91	9.70	0.258
Persistent ST-elevations at discharge, %	27.35	39.28	0.321
Glycoprotein IIb/IIIa inhibitor, %	26.50	48.42	0.070
Aspiration thrombectomy during PCI, %	26.53	51.58	0.041
**Treatment at discharge**			
Acetylsalicylic acid, %	100	100	-
Ticagrelor, %	81.87	60.23	0.094

Clopidogrel, %	18.02	40.34	0.092
B-blocker, %	100	100	-
ACEI/ARB, %	85.27	90.03	0.711
MRA, %	33.39	33.46	1.0
Loop diuretic, %	26.76	40.04	0.323
Statin, %	100	100	-

Table S2. Baseline characteristics of patients with and without reverse remodeling.

STEMI-ST elevation myocardial infarction, NSTEMI-nonST elevation myocardial infarction, GFR-glomerular filtration rate, TIMI-thrombolysis in myocardial infaction, ACEI-angiotensin converting enzyme inhibitor, ARB-angiotensin receptor blocker, MRA-mineralocorticoid receptor antagonist
